# The Discharge Companion Program: An Interprofessional Collaboration in Transitional Care Model Delivery

**DOI:** 10.3390/pharmacy7020068

**Published:** 2019-06-19

**Authors:** Jennifer Bingham, Patrick Campbell, Kate Schussel, Ann M. Taylor, Kevin Boesen, Amanda Harrington, Sandra Leal, Terri Warholak

**Affiliations:** 1 SinfoníaRx, Tucson, AZ 85701, USA; KSchussel@sinfoniarx.com (K.S.); SLeal@sinfoniarx.com (S.L.); 2 Pharmacy Quality Alliance, Alexandra, VA 22315, USA; Pcampbell@pqaalliance.org; 3 College of Pharmacy, University of Arizona, Tucson, AZ 85721, USA; Warholak@pharmacy.arizona.edu (A.M.T.); Taylor@pharmacy.arizona.edu (T.W.); 4 Tabula Rasa Healthcare, Tucson, AZ 85701, USA; Kboesen@TRHC.com; 5 Allergan, Irvine, CA 92612, USA; Amandarharrington@gmail.com

**Keywords:** interprofessional collaboration, pharmacist, nurse, transition-of-care, readmission

## Abstract

To reduce readmission rates and avoid financial penalties from the Centers for Medicare and Medicaid Services, hospitals are seeking to implement innovative transitions of care (TOC) programs. This retrospective study evaluated the Discharge Companion Program (DCP), a pharmacist- and nurse-coordinated interprofessional, collaborative TOC program. Adult patients (18 years and older) from a single hospital, discharged with at least one qualifying diagnosis, were eligible for this service. The hospital transitional care coordinator nurse referred qualified patients to the DCP nurse coordinator, who scheduled telephonic medication therapy management (MTM) reviews with the DCP pharmacist at one- and three-weeks postdischarge. Hospital records and DCP documentation were reviewed to describe respective interventions and assess the impact on 30-day readmissions. A total of 456 patients were referred to the DCP between 31 August, 2015 and 7 September, 2016. Of the 340 patients who participated (DCP group), 44 (13%) compared to 17% (n = 20) of the usual care, were readmitted within 30-days postdischarge. The DCP pharmacists conducted 1242 clinical interventions with participants, demonstrating the benefits of an interprofessional TOC model involving multiple, pharmacist-delivered MTM intervention touchpoints within 30 days post-hospital discharge.

## 1. Introduction

Approximately one in five patients are readmitted post-hospitalization [1]. Hospital readmissions are associated with worsened health outcomes for patients and increased healthcare expenditures [2]. While some readmissions are unavoidable, research suggests that collaborative activities including enhanced patient education, coordination with post-acute care outpatient providers, and reducing medical complications during the patients’ initial hospital stays can prevent readmissions [1].

Reducing readmissions among Medicare beneficiaries is a high priority for the Centers for Medicare and Medicaid Services (CMS), the largest healthcare payer in the United States (US). As such, CMS created the Hospital Readmission Reduction Program (HRRP) to incentivize hospitals to reduce readmission rates [3]. The HRRP financially penalizes hospitals with higher-than-average 30-day readmission rates for select conditions including: myocardial infarction (MI); heart failure; pneumonia; chronic obstructive pulmonary disease (COPD); elective hip or knee replacement; and coronary artery bypass graft (CABG) [3]. Hospitals with readmission rates exceeding the national average are now penalized and receive reduced payments for all Medicare admissions specifically, not just readmissions. According to estimates for 2016 and 2017, more than two thirds of hospitals were slated to receive a financial penalty due to excessive readmission rates. These penalties were substantial and estimated to total $528 million in 2017, $108 million more than the 2016 projections [1]. 

Transition of care (TOC) programs offer a solution to reducing readmission rates. Numerous studies have investigated the impact of telephonic transitional care programs, with varying results [4,5,6,7,8]. Multimodal interventions with patient touch points at different time intervals have shown varying reductions in hospital readmissions [9,10,11,12]. Furthermore, some TOC programs have had medication-related [13] or 30-day postdischarge [14] reductions in emergency department services while others have had statistically significant reductions in readmissions and adverse drug events [15]. A systematic review highlights pharmacists’ major role in successful implementation of the TOC programs. Furthermore, a recent meta-analysis revealed that pharmacy-supported TOC programs are associated with a 40% reduction in the odds of 30-day readmission [16]. 

To date, most programs reported in the literature have been performed by nurses, [4,5,9,11,12,17] pharmacists [6,18] or both [8]. One program provided bedside delivery of postdischarge medications and follow-up phone calls up to 3 days postdischarge; patients who did not receive pharmacist-delivered services had a sixfold increase in the odds of readmission [19]. Another program integrated clinical pharmacist telephonic interventions 2 to 4 days postdischarge and observed lower rates of hospital utilization in the intervention versus usual care group [20]. To our knowledge, no TOC programs have integrated a pharmacist, nurse, and hospital-based transitional care coordinator nurse beyond 5 days postdischarge. Thus, a deficit still exists in the literature regarding the effectiveness of an interprofessional, collaborative approach in providing medication assistance to patients beyond five days postdischarge. The Discharge Companion Program (DCP) was created as a novel program to address this gap in post-discharge services. A retrospective study was conducted to evaluate the impact of the DCP postdischarge.

## 2. Materials and Methods 

### 2.1. Research Design

This retrospective study involved creation and evaluation of the DCP, a program designed to support patients’ transitions of care and reduce hospital readmissions. The Institutional Review Board deemed this a retrospective review (protocol 1707681443; approved 14 August, 2017). 

### 2.2. Methodology

The DCP consisted of an interprofessional pharmacist and nurse, co-coordinated team approach. It provided a comprehensive, expanded focus on multiple conditions highlighted within the HRRP such as renal failure, asthma, and diabetes. The Agency for Healthcare Research on Quality’s (AHRQ) quality improvement initiative goals were also incorporated into the DCP model along with additional emphasis on: improving adherence to national consensus guidelines; a robust medication therapy management (MTM) assessment; and use of health information technology, solely with the intent of reducing all-cause readmissions. 

The DCP partnered with: a single hospital in southern Arizona; and an MTM provider, who provided telephonic, pharmacist-delivered MTM services. Patients were eligible to participate in the DCP if they: were 18 years of age or older; and had a primary discharge diagnosis that included one of these qualifying conditions—asthma, pneumonia, diabetes mellitus, heart failure, chronic obstructive pulmonary disease (COPD), myocardial infarction (MI), total hip or knee replacement, renal failure, or post-coronary artery bypass grafting (CABG). Upon discharge, the hospital’s transitional care coordinator nurse screened patients for the primary discharge conditions during recruitment, taking into account any increased risk of readmission due to advanced age (greater than 80 years), polypharmacy, healthcare literacy and physician referrals. 

If the patient expressed an interest in participating, the transitional care coordinator nurse sent an encrypted secure message to the DCP nurse coordinator within 72 h of discharge. The patient’s preferences regarding the consultation were then noted and communicated to the DCP nurse coordinator. 

If the patient was discharged to a nursing home or rehabilitation facility, the transitional care coordinator nurse notified the case manager at the facility of his/her enrollment in the program and requested that the facility fax the patient’s medication administration record (MAR) to the DCP pharmacist. Ultimately, the transitional care coordinator nurse coordinated with the patient and the patient’s extended care team within 72 h of discharge, prior to the DCP pharmacist contacting the patient. 

**Figure 1:**
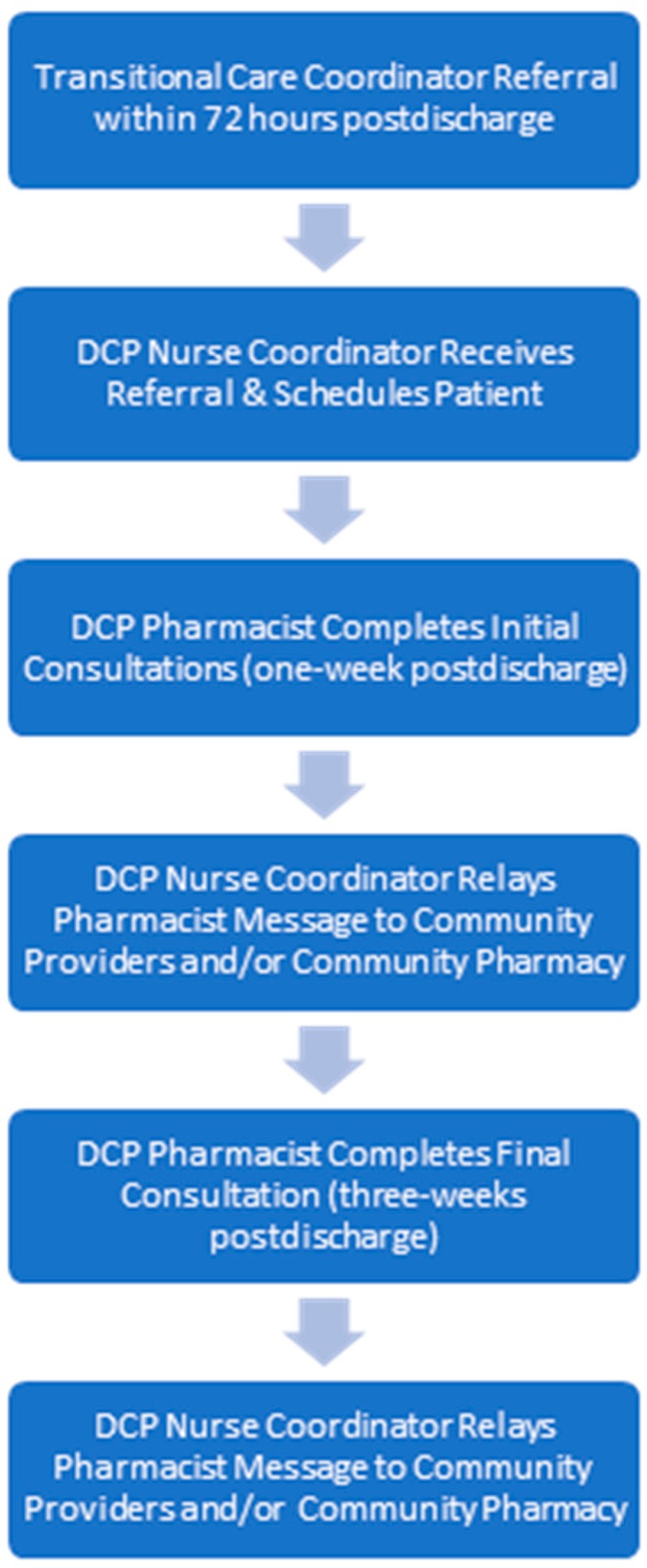
Discharge companion program workflow.

**Figure 2:**
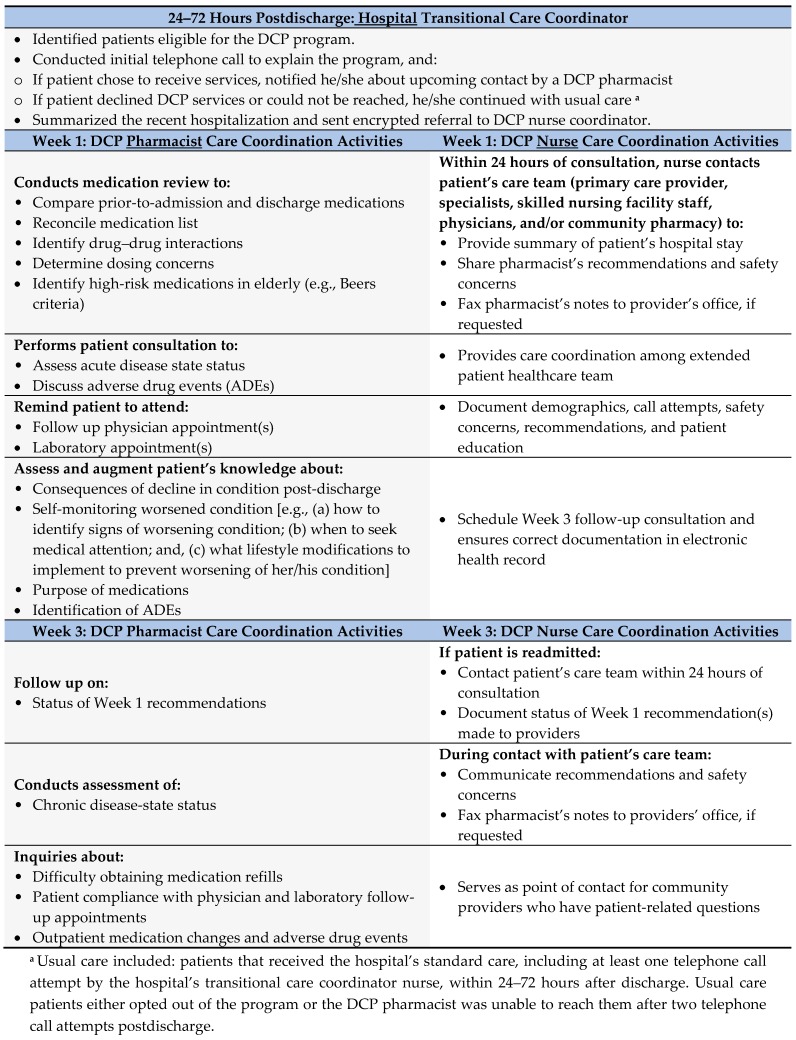
Team member roles and functions during the discharge companion program (DCP).

The initial telephonic patient consultation was performed by the DCP pharmacist within one-week of the patient’s discharge from the hospital; access to the hospital’s electronic health record (EHR) facilitated the pharmacist’s review and documentation of the consultation. Additionally, the MTM staff offered translation services in more than 80 languages, permitting the DCP pharmacist to conduct real-time consultations with a translator and the patient present on the telephone simultaneously. As shown in both Figure 1 and Figure 2, the consultation occurred in one of two settings: (a) patient discharged to home where a DCP pharmacist spoke directly with her/him or the caregiver via telephone; or (b) patient discharged to skilled nursing facility where a DCP pharmacist obtained the facility’s medication administration record, via telephone or facsimile from the facility nurse. 

The DCP pharmacist conducted a medication safety evaluation consisting of these key components including assessment for: (a) therapeutic duplications; (b) drug–disease interactions; (c) drug–drug interactions; (d) dose appropriateness, (e) adverse drug events; and (f) high-risk medication use in the elderly. Barriers to medication adherence, including challenges obtaining new prescriptions, transportation and/or scheduling-related issues with follow-up provider appointments, also were addressed during the direct patient/caregiver consultation. The DCP pharmacist employed teach-back education questions to identify medication nonadherence. 

The DCP also employed teach-back education regarding the discharge diagnosis in an attempt to heighten patient awareness of early symptoms associated with poorer or worsening of patient’s condition, a unique feature of this type of TOC model. Specifically, the patient was prompted, through use of the call script, to determine the extent of her/his knowledge regarding: (a) how to identify signs of worsening condition; (b) when to seek medical attention; and (c) what lifestyle modifications to implement to prevent worsening of her/his condition; the patient then relayed her/his understanding of this information back to the DCP pharmacist. At the end of the consultation, the DCP pharmacist saved the progress note in the hospital’s EHR. 

Within 24 h after the initial pharmacist consultation, the DCP nurse coordinator relayed relevant information to the patient’s primary care provider, specialists, skilled nursing facility staff, physicians, and/or community pharmacy. This information included medication-related recommendations, concerns identified, and any pertinent information related to the patient’s hospitalization. 

The patient was contacted by the DCP pharmacist again three weeks postdischarge. During this week 3 follow-up consultation, the DCP pharmacist addressed the status of any recommendations or concerns identified during the initial telephone call. Additionally, the pharmacist addressed any new issues related to: adherence to national consensus chronic treatment guidelines; cost; general patient questions; medication changes; medication nonadherence; new adverse drug events; and vaccination status. The vaccine-status assessment involved adherence to specific vaccinations, per the Centers for Disease Control and Prevention’ guidelines for influenza, pneumonia, and/or herpes zoster. Following this review, the DCP nurse coordinator again relayed all pertinent information and recommendations to the patient’s providers and/or pharmacy. 

In the interim, the DCP nurse also collected data prospectively during the 30-day period following discharge, maintaining program records for all patients in an internal tracking log. Information captured included patient’s discharge location (i.e., to home alone, home with home healthcare, or to a skilled nursing facility) and noted the contact person for the DCP pharmacist consultation (i.e., patient, a caregiver, or a facility staff member). Additionally, the DCP nurse tracked dates of contact for the 1st- and 3rd-week completed consultations for the intervention group only, or the reason they were not completed (i.e., deceased, readmitted, declined to participate, unable to reach) and the DCP pharmacist’s recommendations or concerns and any problems raised by the patients or other providers. 

### 2.3. Data Collection 

A retrospective review of hospital records and DCP clinical documentation provided the foundation for assessing the impact of the DCP; the review period spanned from 31 August, 2015 to 7 September, 2016. Patients’ hospital EHRs were used to determine 30-day inpatient readmission status to the same hospital. Patients were categorized into two groups based on: participation in the DCP (intervention); or nonparticipation [(usual care (UC)] whereby UC patients either opted out of the program or the DCP pharmacist was unable to reach them after two telephone call attempts postdischarge. Usual care patients received the hospital’s standard care, including at least one telephone call attempt by the hospital’s transitional care coordinator nurse, within 24–72 h after discharge. The hospital’s transitional care coordinator nurse also coordinated the home health, durable medical equipment, and provider appointment(s). 

Clinical interventions made by the DCP pharmacist were tabulated via a retrospective review of the clinical documentation. Interventions were categorized as follows: (a) medication safety evaluation; (b) medication adherence; (c) medication cost saving alternatives; (d) adherence to national consensus therapeutic guidelines; and (e) vaccine adherence. 

A standardized Excel data collection form was developed to capture intervention type (medication safety evaluation, medication adherence, medication cost saving alternatives, adherence to national consensus therapeutic guidelines, vaccine adherence) as well as hospital readmission at the same hospital 30-days postdischarge, group (DCP vs UC), age, gender, ethnicity, race, discharge location (e.g., home or facility), and consultation status (e.g., transitional care coordinator nurse referred patient). The nurse coordinator was trained to enter data directly into the Excel file while reviewing patient records.

### 2.4. Data Analysis

Baseline characteristics of UC and intervention groups were compared using t-tests for continuous variables and chi-square or Fisher’s exact test for categorical variables. A logistic regression model was used to estimate the odds of same hospital readmission at 30-days postdischarge. An adjusted model was run to control for differences in age, gender, ethnicity, race, program qualifying condition, discharge location (i.e., home or facility), and consultation status (i.e., transitional care coordinator nurse referred patient). Odds ratios (OR) and 95% confidence intervals (CI) were reported. An alpha significance level of 0.05 was set a priori. Frequency statistics were used to summarize the DCP pharmacist’s clinical interventions.

## 3. Results

During the program period, a total of 456 hospital patients were referred to the DCP; 75% (n = 340) were included in the intervention group and 25% (n = 116) in the UC group (comparison). As shown in Table 1, intervention group patients were slightly older than the UC patients (mean age [standard deviation, SD]: 77.9 [8.4] vs. 76.1 [9.1] years, *p* < 0.01]). While other characteristics including gender, race, ethnicity, qualifying health conditions, and discharge location were similar between the two groups, DCP participants received consultation (i.e., directed patient referrals) from a hospital transitional care coordinator nurse significantly more often than the UC group (*p* < 0.01).

### 3.1. Thirty-Day Post-Discharge Readmission

Within 30 days of hospital discharge, 13% (n = 44) of the 340 DCP participants and 17% (n = 20) of the 116 UC were readmitted to the same hospital. The odds of readmission did not significantly differ between the DCP and UC groups (OR = 0.56; 95% CI, 0.24–1.30). The adjusted model results were similar to the unadjusted results (AOR = 0.55; 95% CI, 0.25–1.18).

### 3.2. Clinical Interventions

Among the 340 intervention participants (the DCP group), the DCP pharmacist made 1242 clinical interventions. Of the recommendations made: 53% were medication safety related, 2% provided medication cost saving alternatives, 15% pertained to improving national consensus treatment guidelines compliance, 2% targeted improving medication adherence, and 28% pertained to improving vaccine guideline adherence.

There were 190 distinct opportunities identified by the DCP pharmacist to maximize patient care via utilization of guideline recommendations. A majority of the interventions related to medications that patients used for: heart failure (n = 77, 41%); post-MI (n = 44, 23%); post-CABG (n = 20, 11%) and respiratory (asthma and COPD) diseases (n = 41, 22%). A majority of alerts (n = 151, 79.5%) were related to nonadherence to guideline-recommended therapy. For example, there were 39 (21%) occurrences of recommendations to remove a disease-state contraindicated medication. 

The DCP pharmacist also made national vaccine guideline-related recommendations to add 343 immunizations. Recommendations were made for annual influenza (n = 57, 17%), pneumonia (n = 138, 40%) and Herpes Zoster (n = 148, 43%). 

Medication safety concerns (n = 657) comprised the majority of recommendations made by the DCP pharmacist. Medication-safety alert interventions by category included: 29% (N = 193) drug–drug interactions, 23% (N = 150) dose-related concerns, 15% (N = 101) adverse drug reactions, 14% (N = 93) high-risk medication use, 12% (N = 81) drug–disease interactions, and 6% (N = 39) therapeutic duplication alerts. The DCP pharmacist also addressed 22 barriers to adherence and recommended a cost-saving therapy alternative for intervention patients on 30 distinct occasions. 

## 4. Discussion

The DCP provided multiple patient touchpoints postdischarge, facilitated by the pharmacist’s access to the patient’s EHR, enabling integration of this information in to the consultation recommendations made. As a result, the DCP pharmacist provided a wide variety of clinical interventions, with the majority related to improving medication safety (53%) and adherence to treatment guidelines for medications and vaccines (43%). Clinical interventions, such as those made during the DCP, have the potential to improve overall patient safety and reduce healthcare-related costs. The DCP results suggest that pharmacists can effectively deliver post-hospital discharge clinical services to patients while simultaneously helping to improve medication safety and guideline compliance. However, further research is needed to investigate the associated patient-related health outcomes.

The current study findings are consistent with others’ research on integration of pharmacists on the TOC team [6,14,21]. However, the DCP is unique among TOC programs given that it utilizes nurses and pharmacists in conjunction with the hospital’s transitional care coordinator nurse. Moreover, the DCP nurse and pharmacist continued to coordinate with the hospital transitional care coordinator nurse post-patient discharge. 

While other studies have focused on psychosocial issues; [4,7,8] disease-state education and basic adherence advice; [4,5,6,8,9,11,17,18] and medication management [6,8,18], a distinctive component of this study was the ongoing interprofessional communication. Rochester-Eyeguokan et al. found that multimodal, multidisciplinary interventions with an emphasis on strengthening communication systems were the most effective TOC strategy [21]. Similar to the findings of Rochester-Eyeguokan et al., the reduced readmissions in the current study suggest that sustained interprofessional communication was a potential benefit in helping reduce patient readmissions. Furthermore, the DCP integrated additional communication components with the patient’s providers and his/her community pharmacy. 

Multiple touchpoints and bundling of interventions (e.g., medication reconciliation, multi-faceted interventions) have shown promise [22,23] and EHR access has led to more comprehensive and informed recommendations [24]. The DCP’s interprofessional, multiple touchpoint interventions parallel other effective TOC programs reported by Rochester-Eyeguokan et al. [21]. Specifically, all of these components were integrated into the DCP, making it to date, one of the most comprehensive, interprofessional and communicative models described in the literature. 

The association between the DCP and readmission to only one hospital showed no significant difference in the rate, in either the adjusted or unadjusted analyses. While the adjusted analysis controlled for certain variables, it was impossible to account for all confounders (e.g., previous hospital admissions), making it difficult to detect significant differences. Furthermore, it is plausible that this nonsignificant finding is attributable to inadequate power and lack of important covariates (e.g., prior admission status). Other TOC programs have shown a significant impact on readmission [4,5,6,8,9,11,17] and several have analyzed readmissions to more than one hospital or all hospitals [4,5,6,7,8,9,11,17,18]. Additionally, a systematic review suggested that 30 days may be too soon to see the full impact of multi-faceted interventions [23]. Future research is needed to: include data on all-hospital readmissions (rather than one hospital only) including 30-day readmission rates prior to program implementation; evaluate TOC programs in larger patient populations; and assess their impact on a larger scale and the program’s return-on-investment. 

Based on the DCP’s results, a similar model may prove appropriate for implementation at the health-plan level. The structured and standardized approach to providing care for each patient allows for program replication in other patient populations and various healthcare settings while the program evaluation provides a mechanism for regular review and continual process improvement. However, future work may warrant identification of essential hospital and DCP personnel to realistically enable implementation in other practice settings. Additionally, more work is needed to monitor outcomes related to the non-hospital (in this case the DCP nurse) nurse’s involvement. Furthermore, additional analyses comparing readmission rates excluding patients receiving home health and/or other skilled nursing services could provide additional insight. This program was reliant on like-minded, innovative hospitals, health systems and payers to provide reimbursement for this MTM model. As CMS models move to episodic payment rather than fee-for-service, providers will presumably have financial incentives to better coordinate care to prevent readmissions.

### Limitations

This project had several limitations. First, subjects were not randomized to study groups. Patients participated in the DCP on a voluntary basis, potentially leading to self-selection bias. Second, it was impossible to control for confounding variables that were not collected during the study period, such as previous hospital admissions, thus limiting the ability to detect differences between groups. Third, the DCP evaluated readmission data from only one hospital. This is important because patients may have been readmitted for the same condition to other non-participating hospitals and thus, this information was not captured for the present analysis. Moreover, future work necessitates evaluation of 30-day readmissions prior to program implementation for comparison between groups (intervention, control). Fourth, access to the local hospital’s prescription and Medicare claims data was not granted nor included in the DCP analysis, thus provider acceptance rates and patient adherence were not calculated. 

## 5. Conclusions

The DCP results on 30-day hospital readmissions to the same hospital were not significant. However, the breadth of medication-related interventions implemented illustrates the potential benefits of an interprofessional, collaborative TOC model with multiple pharmacist-delivered MTM touchpoints within 30 days of hospital discharge. Future work is warranted to assess the impact of the DCP’s unique interventions on all-hospital readmissions and its associated return on investment. Finally, implementation of the essential hospital- and DCP-based program components may provide valuable insight in other practice settings. 

## Figures and Tables

**Table 1:** Demographic characteristics of usual care group and Discharge Companion Program (DCP) participants (intervention group).

Characteristic	UC Group (N = 116)	Intervention Group (N = 340)	*p*-Value
**Age, mean (SD)**	76.1 (9.1)	77.9 (8.4)	<0.04 *
**Male, n (%)**	73 (63)	203 (60)	0.54
**White, n (%)**	107 (92)	318 (93)	0.61
**Hispanic, n (%)**	19 (16)	59 (17)	0.94
**Program qualifying conditions ^1^**			0.14
**Asthma**	1 (1)	6 (2)	
**Chronic obstructive pulmonary disease**	11 (9)	34 (10)	
**Diabetes mellitus**	0 (0)	2 (1)	
**Heart failure**	50 (43)	121 (36)	
**Myocardial infarction**	20 (17)	53 (16)	
**Pneumonia**	26 (22)	61 (18)	
**Post-coronary artery bypass graft**	6 (5)	61 (18)	
**Renal failure**	2 (2)	2 (1)	
**Received transitional care coordinator nurse referral to DCP pharmacist, n (%)**	49 (42)	201 (59)	<0.01 *
**Discharged to a facility, n (%)**	36 (31)	91 (27)	0.46
**All-cause readmission rate, n (%)**	20 (17)	44 (13)	0.29

SD = standard deviation; UC = usual care. ^1^ Primary discharge condition that qualified the patient for referral into the program. * Statistically significant difference between groups based on t-tests for continuous data; chi-square or fisher’s exact test for categorical data.
